# CXCL8 and CCL20 Enhance Osteoclastogenesis via Modulation of Cytokine Production by Human Primary Osteoblasts

**DOI:** 10.1371/journal.pone.0131041

**Published:** 2015-06-23

**Authors:** Janak L. Pathak, Astrid D. Bakker, Patrick Verschueren, Willem F. Lems, Frank P. Luyten, Jenneke Klein-Nulend, Nathalie Bravenboer

**Affiliations:** 1 Department of Oral Cell Biology, Academic Centre for Dentistry Amsterdam (ACTA), University of Amsterdam and VU University Amsterdam, MOVE Research Institute Amsterdam, Amsterdam, The Netherlands; 2 Skeletal Biology and Engineering Research Center, KU Leuven, Leuven, Belgium; 3 Department of Rheumatology, VU University Medical Center, MOVE Research Institute Amsterdam, Amsterdam, The Netherlands; 4 Department of Clinical Chemistry, VU University Medical Center, MOVE Research Institute Amsterdam, Amsterdam, The Netherlands; Faculté de médecine de Nantes, FRANCE

## Abstract

Generalized osteoporosis is common in patients with inflammatory diseases, possibly because of circulating inflammatory factors that affect osteoblast and osteoclast formation and activity. Serum levels of the inflammatory factors CXCL8 and CCL20 are elevated in rheumatoid arthritis, but whether these factors affect bone metabolism is unknown. We hypothesized that CXCL8 and CCL20 decrease osteoblast proliferation and differentiation, and enhance osteoblast-mediated osteoclast formation and activity. Human primary osteoblasts were cultured with or without CXCL8 (2–200 pg/ml) or CCL20 (5–500 pg/ml) for 14 days. Osteoblast proliferation and gene expression of matrix proteins and cytokines were analyzed. Osteoclast precursors were cultured with CXCL8 (200 pg/ml) and CCL20 (500 pg/ml), or with conditioned medium (CM) from CXCL8 and CCL20-treated osteoblasts with or without IL-6 inhibitor. After 3 weeks osteoclast formation and activity were determined. CXCL8 (200 pg/ml) and CCL20 (500 pg/ml) enhanced mRNA expression of *KI67* (2.5–2.7-fold), *ALP* (1.6–1.7-fold), and IL-6 protein production (1.3–1.6-fold) by osteoblasts. CXCL8-CM enhanced the number of osteoclasts with 3–5 nuclei (1.7-fold), and with >5 nuclei (3-fold). CCL20-CM enhanced the number of osteoclasts with 3–5 nuclei (1.3-fold), and with >5 nuclei (2.8-fold). IL-6 inhibition reduced the stimulatory effect of CXCL8-CM and CCL20-CM on formation of osteoclasts. In conclusion, CXCL8 and CCL20 did not decrease osteoblast proliferation or gene expression of matrix proteins. CXCL8 and CCL20 did not directly affect osteoclastogenesis. However, CXCL8 and CCL20 enhanced osteoblast-mediated osteoclastogenesis, partly via IL-6 production, suggesting that CXCL8 and CCL20 may contribute to osteoporosis in rheumatoid arthritis by affecting bone cell communication.

## Introduction

Generalized osteoporosis is common in patients with systemic inflammatory disease such as rheumatoid arthritis (RA) and Crohn’s disease [1, 2]. RA is a systemic, autoimmune inflammatory disease of unknown etiology characterized by chronic inflammation [3]. Hallmarks of RA are local bone erosion and joint space narrowing, but extra-articular manifestations such as generalized osteoporosis are also common [1, 3]. Local and generalized bone loss results from an imbalance in osteoblastic bone formation and osteoclastic bone resorption during bone remodeling. Unfortunately the underlying mechanisms of this imbalance are not fully elucidated.

The cause of bone loss during systemic inflammation is multifactorial, such as lack of physical activity, use of corticosteroids, and increased levels of inflammatory cytokines [4]. Serum from patients with active RA contain circulating factors, likely cytokines and chemokines, that inhibit osteoblast proliferation and differentiation, and modulate endogenous cytokine production by osteoblasts, thereby affecting osteoclastogenesis [5]. Chronic inflammation in RA enhances production of proinflammatory cytokines such as tumor necrosis factor-α (TNF-α), interleukin-1β (IL-1β), and interleukin-6 (IL-6), as well as production of chemokines such as CXCL8, CXCL9, CXCL10, and CCL20 in arthritic joints [6–8]. During bone remodeling, osteoblasts and osteocytes also release cytokines, e.g. receptor activator of nuclear factor-kappa B ligand (RANKL), osteoprotegerin (OPG), IL-1β, and IL-6 [9, 10]. Cytokines, such as TNF-α and IL-1β, affect osteoblastic cytokine production in an autocrine manner [9, 10]. TNF-α, RANKL, OPG, IL-1β, and IL-6 play a vital role in osteoclast formation and activity [9–11]. Enhanced levels of proinflammatory cytokines such as TNF-α, IL-1β, IL-6, and IL-17 in arthritic joints and in the systemic circulation do not only directly disturb the balance between osteoblastic bone formation and osteoclastic bone resorption [12, 13], but also affect osteocyte and/or osteoblast communication towards osteoclasts resulting in bone loss [14, 15]. This indicates a possible role of cytokines/chemokines in bone loss during systemic inflammation.

Chemokines are small (8–12 KD) chemo-attractant cytokines, which bind G-protein–coupled receptors [16, 17]. Chemokines play an important role in immunological tolerance and movement of immune cells [16]. Although chemokines are involved in the pathogenesis of RA [7], their role in bone remodeling is still unclear. Indeed, elevated levels of CXCL8, CXCL9, CXCL10, and CCL20 have been demonstrated in the synovium and serum of RA patients, and receptors for these chemokines have been detected in human primary osteoblasts, i.e. the receptor for CXCL8 (CXCR1), CXCL9, CXCL10 (CXCR3), and CCL20 (CCR6) [18–22]. Moreover osteoblasts from RA patients express more CCL20 and CCR6 than osteoblasts from patients with osteoarthritis or healthy individuals. Osteocytes and mononuclear cells from RA patients also express CCL20 and CCR6 [20, 23]. Thus CXCL8, CXCL9, CXCL10, and CCL20 could contribute to bone loss in inflammation, which might provide novel targets for intervention in patients with inflammatory disease, when inhibition of other cytokines such as IL-6 and TNF-α does not suffice to restore bone remodeling to normal levels.

The effect of cytokines on bone remodeling is well described, but much less is known about the effect of chemokines on bone remodeling [14]. Until now, no data are available concerning the role of chemokines in localized and generalized osteoporosis in RA or other inflammatory diseases. Therefore we hypothesized that elevated levels of chemokines play a role in osteoporosis in RA. We aimed to analyze the effect of CXCL8, CXCL9, CXCL10, and CCL20 on human primary osteoblast proliferation, gene expression of matrix proteins, and osteoblast-mediated osteoclast formation and activity. Our data indicate a role for CXCL8 and CCL20 in osteoclastogenesis through their effect on osteoblasts.

## Materials and Methods

### Osteoblast Culture

Trabecular bone samples (surgical waste) from 4 female and 3 male donors (63.3±7.8 yrs; range 53–75 yrs) were obtained from the iliac crest during sinus floor elevation surgery using autologous bone graft. Serum C-reactive protein (CRP) levels of all donors was <2.5 mg/l, indicating no inflammatory disease. The protocol was approved by the Ethical Review Board of the VU University Medical Center and all subjects gave written informed consent.

Osteoblast cultures were established as described earlier [24]. Briefly, trabecular bone fragments were placed in sterile phosphate-buffered saline (PBS), chopped into small fragments, and washed extensively with PBS. Bone fragments were then incubated with 2 mg/ml collagenase type II (Worthington, Freehold, NJ) in Dulbecco’s Modified Eagle’s Medium (DMEM; Gibco, Grand Island, NY)/Nutrient mixture F-12 (F-12) (DMEM/F-12, 1:1 (vol/vol)) for 2 h at 37°C in a shaking water bath to remove all adhering cells from the bone fragment surfaces. Bone fragments were then washed with medium containing 10% Fetal Clone I serum (HyClone), subdivided into equal portions, and transferred to culture flasks (Nunc, Roskilde, Denmark). To obtain outgrowth of bone cells, bone fragments were cultured in DMEM/F-12 supplemented with 10% Fetal Clone I serum, 100 U/ml penicillin (Sigma, Hamburg, Germany), 100 μg/ml streptomycin sulfate (Gibco), 50 μg/ml gentamicin (Gibco), 1.25 μg/ml fungizone (Gibco), and 100 μg/ml ascorbate (Merck, Darmstadt, Germany) at 37°C in a humidified atmosphere with 5% CO_2_. The culture medium was refreshed twice a week until cells reached confluence. After reaching confluency, cells were trypsinized and seeded at 1x10^4^ cells/well of 24-well culture plates (Greiner Bio-One, Frickenhausen, Germany), incubated overnight, and treated with or without chemokines as described below.

### Flow Cytometric Analysis of Functional Chemokine Receptors on Osteoblasts

Osteoblasts obtained from bone biopsies of 3 donors (two females: 53 and 58 yrs, and one male: 66 yrs) were used to analyze the expression of chemokine receptors. Osteoblasts were harvested at 80–90% confluency. Cells were resuspended in phosphate-buffered saline (PBS) with 0.1% bovine serum albumin and 0.1% sodium azide for FACS analysis. For cell surface receptor antigen detection, cells were incubated with fluoro-isothiocyanate (FITC) or phycoerythrin (PE)-conjugated antibodies. 5x10^4^ cells were incubated for 30 min at 4°C with human antibodies CD44-PE (G44-26, IgG2b), CD44-FITC (G44-26, IgG2b), CCR6-PE (11A9, IgG1), CXCR1-PE (5A12, IgG1), CXCR3-PE (1C6/cxcr3, IgG1). CD44^+^ cells were gated using CD44-PE and CD44-FITC antibody. Chemokine receptor expression was further analyzed in the CD44^+^ cell population on a FACScan with Cell Quest software (Becton Dickinson Immunocytometry Systems, Mountain View, CA). All antibodies used were from BD Pharming (San Diego, CA). To analyze the functionality of CXCR1 and CCR6, osteoblasts were cultured for 48 h with the chemokine ligand CXCL8 (20 pg/ml) for CXCR1 and CCL20 (50 pg/ml) for CCR6. Chemokine-treated osteoblasts were then analyzed again for CXCR1 and CCR6 expression.

### Chemokine Treatment

Osteoblasts obtained from bone biopsies from 4 different donors (two females: 59 and 61 yrs, and two males: 71 and 75 yrs) were used to analyze the effect of CXCL8 and/or CCL20, and TNF-α on osteoblast proliferation and differentiation, and osteoblast-to osteoclast communication. Osteoblasts seeded in 24 well-culture plates were incubated overnight and treated with or without recombinant human CXCL8 (2, 20, 200 pg/ml; Sigma), CCL20 (5, 50, 500 pg/ml; Sigma), a combination of CXCL8 (20 pg/ml) + CCL20 (50 pg/ml), or TNF-α (100 ng/ml; Sigma) for 14 days. The effect of CXCL8, CCL20, CXCL8+CCL20, or TNF-α on osteoblast proliferation and differentiation was analyzed. Chemokine concentrations tested were chosen based on RA-serum concentrations. Concentrations which were 10 times lower or higher than the levels reported in RA-serum were also tested [7, 8]. TNF-α was used as positive control. Chemokine treatment was performed for 14 days to mimic *in vivo* chronic inflammation as in RA. CM from osteoblasts cultured for 14 days with control medium (control-CM), CXCL8 at 2, 20, and 200 pg/ml (CXCL8-CM), CCL20 at 5, 50, and 500 pg/ml (CCL20-CM), CXCL8 + CCL20 (CXCL8+CCL20-CM) and TNF-α (TNF-α-CM) were collected.

### Osteoblast Proliferation

Proliferation of osteoblasts was tested in cells seeded at 2.5x10^3^ cells/well of 96-well culture plates (Greiner Bio-One). The following day, medium was replaced by fresh medium with or without CXCL8, CCL20, CXCL8+CCL20, or TNF-α. Cell proliferation was determined after 3, 5, and 7 days of culture using a Cell Proliferation Kit II (XTT; Roche, Mannhelm, Germany).

The total DNA content of the cell layer was quantified using a Cyquant Cell Proliferation Assay (Molecular Probes, Eugene, OR).

### Procollagen Type 1 Amino-terminal Propeptide (P1NP) and Alkaline Phosphatase (ALP) Activity

P1NP in CM and ALP in cell lysate of osteoblast cultures were measured as described earlier [25].

### RNA Isolation and Real-time RT-PCR

Total RNA of primary osteoblasts was isolated using an RNeasy Micro kit with an on-column DNase I digestion (Qiagen, Basel, Switzerland). Total RNA concentrations were measured with a Nanodrop spectrophotometer (Nanodrop Technologies, Wilmington, DE). cDNA synthesis was performed in a thermocycler GeneAmp PCR System 9700 PE (Applied Biosystems, Foster City, CA), using a SuperScript VILO cDNA Synthesis Kit (LifeTechnologies, Inchinnan, UK), with 0.1 μg of total RNA in 20 μl reaction mixture consisting of VILO Reaction Mix and SuperScript Enzyme Mix.

Real-time PCR reactions were performed using 2.5 μl cDNA and SYBR Green Supermix (Roche Laboratories, Indianapolis, IN) in a LightCycler (Roche Diagnostics). In each PCR run, the reaction mixture without cDNA was used as a negative control. For quantitative real-time PCR, the values of relative target gene expression were normalized for relative mean of *YWHAZ* and *HPRT* housekeeping gene expression. Real-time PCR was used to assess expression of the following genes: *KI67*, collagen 1 (*COL1*), *ALP*, osteopontin (*OPN*), osteocalcin (*OCN*), macrophage colony stimulating factor (*MCSF*), *RANKL*, *OPG*, *IL-1β*, *IL-6*, *IL-17*, *CXCL8*, *CCL20*, and cysteine rich protein 61 (*CYR61*). All primers used were from LifeTechnologies. The primer sequences are listed in S1 Table.

### IL-6 Protein Quantification

CM was collected from osteoblasts after 14 days of culture in the presence or absence of chemokines, and IL-6 protein was quantified using a PeliKine human IL-6 ELISA kit (Sanquin Blood Supply, Amsterdam, Netherlands).

### Osteoclastogenesis

Peripheral blood mononuclear cells (PBMCs) were isolated from a buffy coat (Sanquin) as described previously [26]. Buffy coats were obtained from blood donated by healthy blood donors at Sanquin Blood Supply, Amsterdam, The Netherlands. PBMCs were seeded at 5x10^5^ cells/well of 96 well plates or on bovine bone slices in DMEM containing 10% FCS, antibiotics, and control-CM, CXCL8-CM from 200 pg/ml CXCL8 treatment, CCL20-CM from 500 pg/ml CCL20 treatment, CXCL8+CCL20-CM, and TNF-α-CM (ratio DMEM:CM = 1:1 (v/v)). Twenty-five ng/ml recombinant human M-CSF (R&D Systems, Minneapolis, MN) was added to the cells from day 1 to day 3. Ten ng/ml M-CSF and 4 ng/ml human RANKL (Peprotech, London, UK) were added from day 3 to day 21. To similar cultures, 0.15 μg/ml human IL-6 antibody (Clone #6708, R&D Systems) was added and IgG isotype control (Clone #11711, R&D Systems) was used as control for IL-6 antibody. PBMCs were also cultured with DMEM containing 10% FCS, antibiotics, and either CXCL8 (200 pg/ml) or CCL20 (500 pg/ml). After 3 weeks, cells were fixed in 4% formaldehyde, and stained for tartrate-resistant acid phosphatase (TRACP; Sigma). Nuclei were visualized by 4’,6-diamidino-2-phenylindole (DAPI) staining. Osteoclastogenesis was assessed by counting the number of TRACP-positive osteoclasts containing >3 nuclei per cell on 10 pre-determined microscopic fields in the each well.

### Osteoclastic Bone Resorption

To quantify osteoclast activity, resorption pits in bone slices were visualized and counted as described earlier [27]. The resorbed area was measured using Image Pro-Plus Software (Media Cybernetics, Silver Spring, MD) and expressed as percentage of total bone surface area. The percentage of bone resorption was expressed per number of TRACP-positive multinucleated cells.

### Statistical Analysis

Data on *KI67* gene expression, TRACP-positive multinucleated osteoclast number, and osteoclastic bone resorption was expressed as mean±SEM. Data on expression of other genes analyzed and IL-6 protein production were expressed as median with 5–95 percentile range of the treatment-over-control ratio. Differences in gene expression and IL-6 protein production between chemokine-treated and untreated control cultures were tested using the Wilcoxon Signed Rank test. The effect of control-CM or chemokine-CM on osteoclastic bone resorption, and the effect of control-CM or chemokine-CM and CM+IL-6 inhibitor on osteoclast formation was tested using ANOVA followed by Bonferroni’s Multiple Comparison test. Differences were considered significant if p<0.05. Statistical analysis was performed using GraphPad Prism 5.01 (GraphPad Software, Inc., La Jolla, CA, USA).

## Results

### Osteoblasts Expressed CXCL8 and CCL20 Receptors

**Fig 1:**
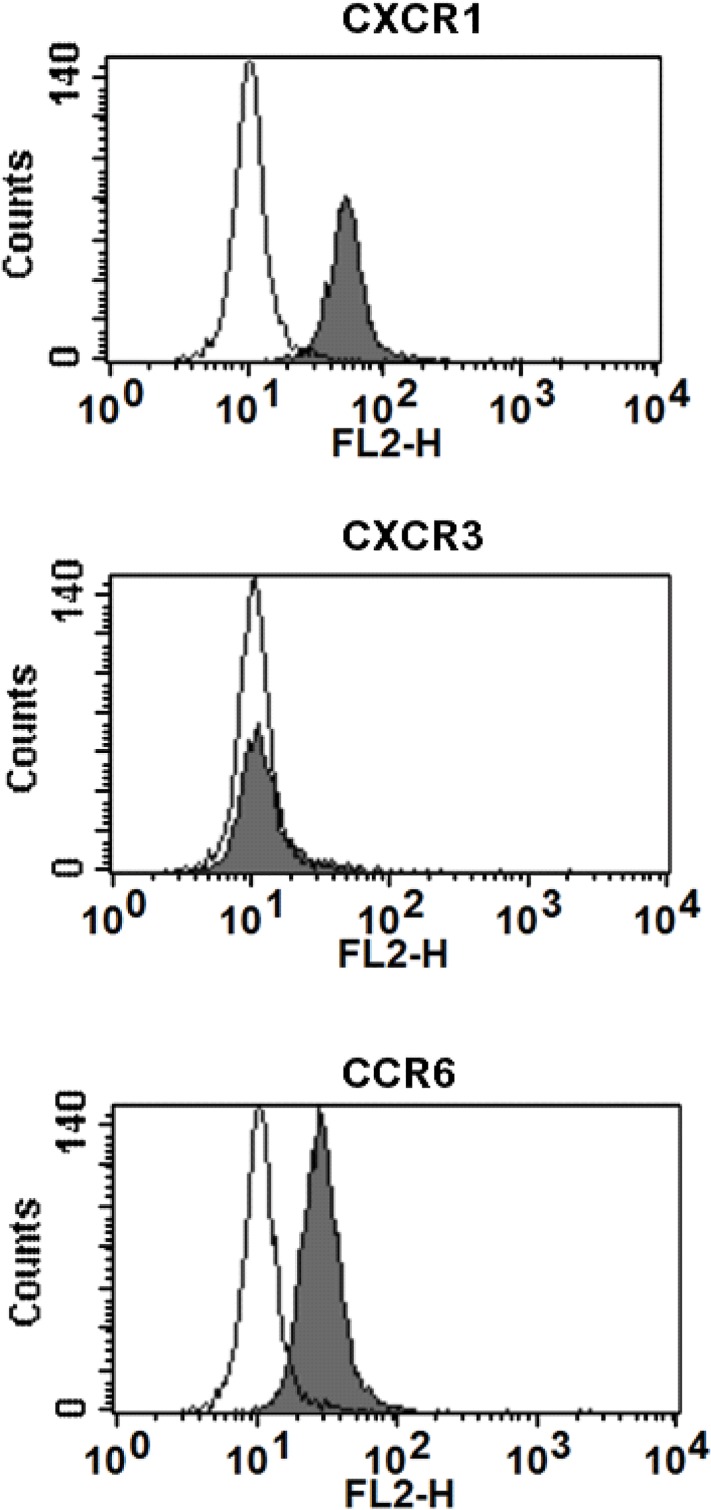
Osteoblasts expressed CXCR1 and CCR6 receptors. Flow cytometric analysis of CXCR1, CXCR3 and CCR6 receptor expression by primary bone cells. Open histogram: isotype control; shaded histogram: chemokine receptor antibody.

Osteoblasts were highly positive for chemokine receptor CXCR1 (72.4±7.7%, mean±SEM), and to a lesser extent for CCR6 (15.6±8.2%; Fig 1). Osteoblasts did not express CXCR3. After 48 h of culture with chemokines, CXCR1 receptor expression decreased by 70% and CCR6 expression by 64% (Table 1). Since osteoblasts did not express the chemokine receptor CXCR3 for CXCL9 and CXCL10, these chemokines were not used for further study.

**Table 1:** Functional chemokine receptor expression in human primary bone cells after 48 h of culture with or without CXCL8 (200 pg/ml) or CCL20 (500 pg/ml).

	% Cells expressing receptor	
	Without CXCL8 or CCL20	With CXCL8 or CCL20
CXCR1	72.4 ± 7.7	21.2 ± 6.4
CCR6	15.0 ± 8.2	5.6 ± 1.8

Values are mean ± SD of the percent of human primary bone cells expression CXCR1 or CCR6. n = 3 experiments, using cells obtained from 3 independent donors.

### CXCL8 and CCL20 Did Not Inhibit Proliferation Nor Gene Expression of Matrix Proteins in Osteoblasts

**Fig 2:**
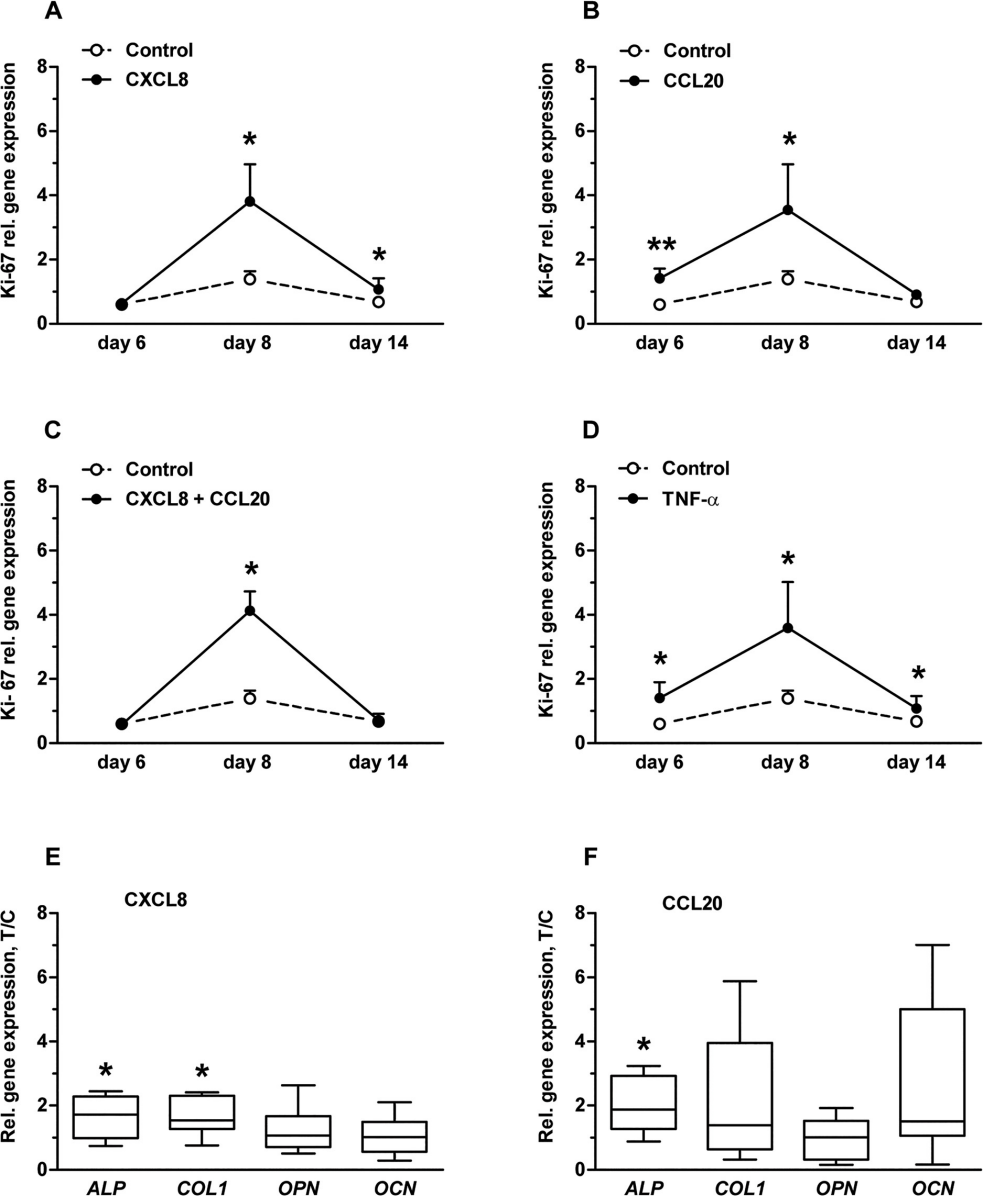
CXCL8 (2, 20, and 200 pg/ml) and CCL20 (5, 50, and 500 pg/ml) enhanced Ki-67 gene expression, but only 200 pg/ml of CXCL8 and 500 pg/ml of CCL20 enhanced early osteogenic marker gene expression in osteoblasts. **(A)** CXCL8 (200 pg/ml) enhanced Ki-67 expression (day 8,14). **(B)** CCL20 (500 pg/ml) enhanced ki-67 expression (day 6,8). **(C)** CXCL8+CCL20 (20 pg/ml+50 pg/ml) enhanced ki-67 expression (day 8). **(D)** TNF-α (100 ng/ml) enhanced Ki-67 expression (day 6,8,14). **(E)** CXCL8 (200 pg/ml) enhanced ALP and COL1 expression (day 14). **(F)** CCL20 (500 pg/ml) enhanced ALP expression (day 14). Values are median with 5–95 percentile range of treatment-over-control ratios from 3 experiments, n = 9. Significant effect of chemokines or TNF-α, *p<0.05, **p<0.01.

CXCL8 (200 pg/ml) increased *KI67* gene expression by 2.7-fold at day 8 and 1.6-fold at day 14 (Fig 2A). CCL20 (500 pg/ml) increased *KI67* expression by 2.4 fold at day 6 and 2.5-fold at day 8 (Fig 2B). The other CXCL8 and CCL20 concentrations tested did not affect *KI67* expression. The combination of CXCL8+CCL20 (20 pg/ml+50 pg/ml) enhanced *KI67* expression by 3.7-fold at day 8 (Fig 2C). TNF-α enhanced *KI67* expression by 2.3, 2.6, and 1.6-fold at day 6, 8, and 14 respectively (Fig 2D). CXCL8 and/or CCL20 treatment did not affect osteoblast proliferation as measured by the XTT assay, nor the total DNA content (data not shown).

CXCL8 (200 pg/ml) increased gene expression of differentiation marker *ALP* by 1.7-fold, and *COL1* by 1.5-fold at day 14 (Fig 2E). CCL20 (500 pg/ml) increased *ALP* expression by 1.6-fold (Fig 2D). CXCL8 (200 pg/ml) and CCL20 (500 pg/ml) did not affect gene expression of the matrix proteins *OPN* and *OCN*. Neither CXCL8 at 2 or 20 pg/ml, CCL20 at 5 or 50 pg/ml, nor TNF-α (100 ng/ml) or CXCL8+CCL20 (20 pg/ml+50 pg/ml) did affect expression of *COL1*, *OPN*, *or OCN*. P1NP formation and ALP activity was not changed by chemokines or TNF-α (data not shown).

### CXCL8 and CCL20 Enhanced *IL-6* Gene Expression and IL-6 Protein Production by Osteoblasts

**Fig 3:**
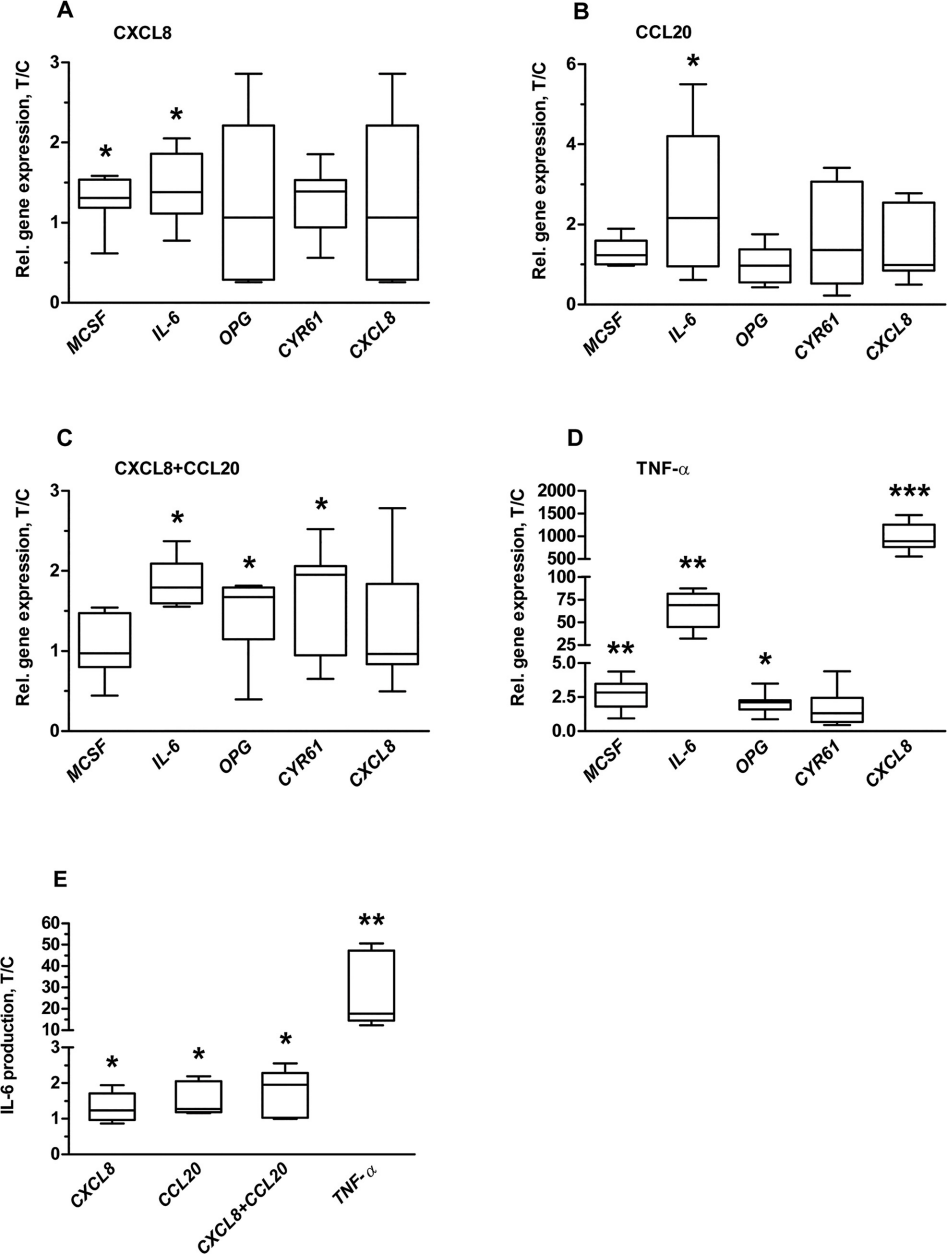
CXCL8 (200 pg/ml) and CCL20 (500 pg/ml) affected osteoblast-to-osteoclast communication at day 14. **(A)** CXCL8 (200 pg/ml) enhanced MCSF and IL-6 gene expression. **(B)** CCL20 (500 pg/ml) enhanced IL-6 gene expression. **(C)** CXCL8+CCL20 (20 pg/ml+50 pg/ml) enhanced IL-6, OPG, and CYR61 gene expression. **(D)** TNF-α (100 ng/ml) enhanced MCSF, IL-6, and OPG gene expression. **(E)** CXCL8 (200 pg/ml), CCL20 (500 pg/ml), CXCL8+CCL20 (20 pg/ml+50 pg/ml), and TNF-α (100 ng/ml) enhanced IL-6 production by osteoblasts. Values are median with 5–95 percentile range of treatment-over-control ratios from 3 experiments, n = 9. Significant effect of chemokines and TNF-α, *p<0.05, **p<0.01.

CXCL8 (200 pg/ml) increased gene expression of MCSF by 1.3-fold and *IL-6* by 1.4-fold at day 14 (Fig 3A). CCL20 (500 pg/ml) increased *IL-6* expression by 1.6-fold (Fig 3B). The combination of CXCL8+CCL20 (20 pg/ml+50 pg/ml) increased expression of *IL-6* by 1.7-fold, *OPG* by 1.4-fold, and *CYR61* by 1.7-fold (Fig 3C). TNF-α (100 ng/ml) increased expression of *MCSF* by 2.8-fold, *IL-6* by 64-fold, *OPG* by 2.1-fold, and CXCL8 by 836-fold (Fig 3D). Neither CXCL8 at 2 or 20 pg/ml, nor CCL20 at 5 or 50 pg/ml did affect cytokine gene expression by osteoblasts (data not shown). Since only IL-6 gene expression was enhanced by CXCL8 (200 pg/ml), CCL20 (500 pg/ml), CXCL8+CCL20 (20 pg/ml+50 pg/ml), and TNF-α (100 ng/ml) treatment in osteoblasts, we measured IL-6 protein production in the CM from osteoblasts. CXCL8 (200 pg/ml), CCL20 (500 pg/ml), CXCL8+CCL20 (20 pg/ml+50 pg/ml), and TNF-α (100 ng/ml) increased IL-6 production by 1.3, 1.6, 2.2, and 29-fold respectively compared to controls (Fig 3E). The mean IL-6 concentration in control CM was 148 pg/ml. Gene expression of *TNF-α*, *RANKL*, *IL-1β*, *CCL20*, and *IL-17* was below the detection limit (ct value >38).

### CXCL8 and CCL20 Enhanced Osteoclastogenesis Partly via IL-6 Production by Osteoblasts

**Fig 4:**
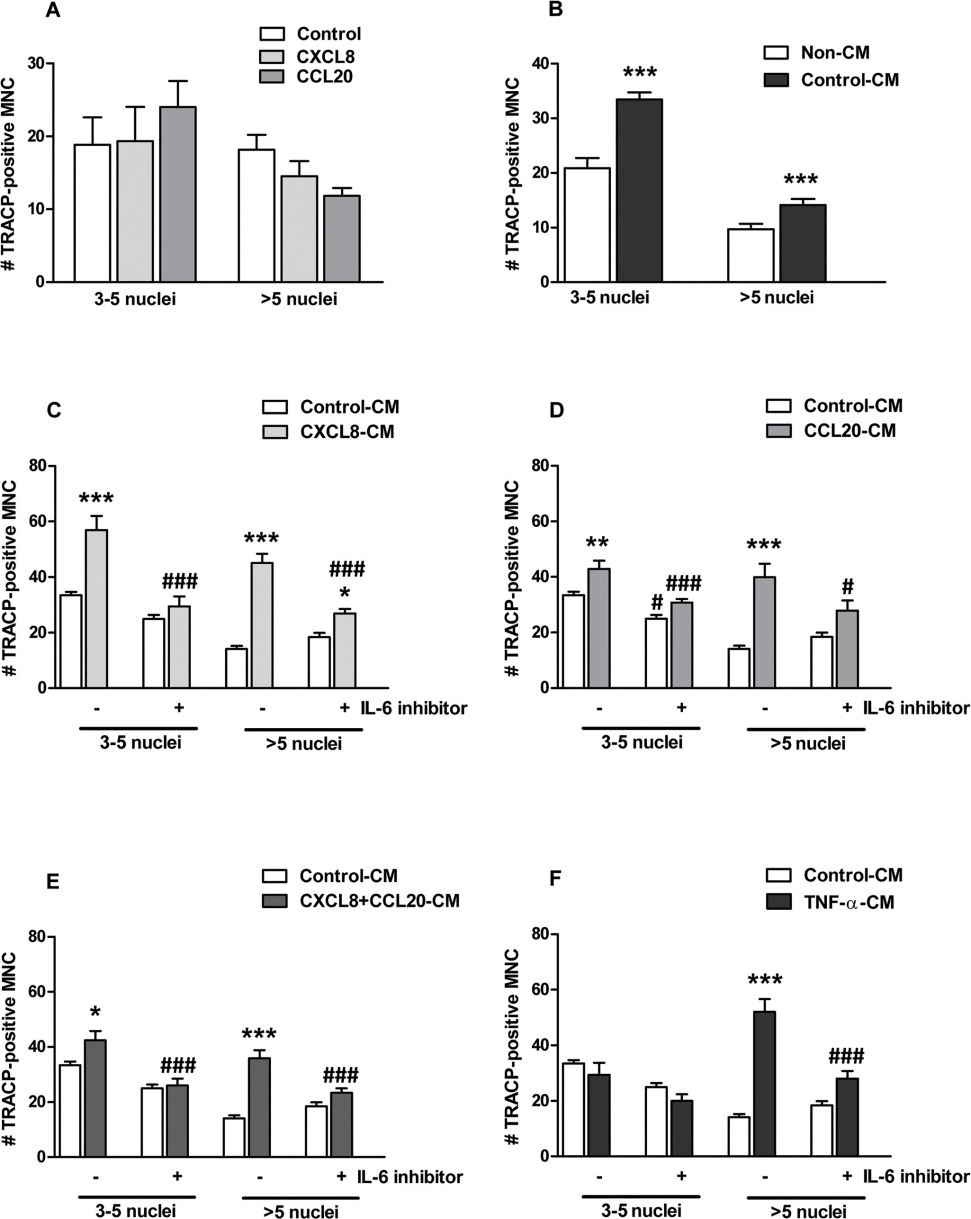
Effect of CXCL8 (200 pg/ml), CCL20 (500 pg/ml), CXCL8-CM, and CCL20-CM on osteoclast formation. (**A)** CXCL8 (200 pg/ml) and CCL20 (500 pg/ml) did not affect TRACP-positive multinucleated cell (TRACP^+^ MNC) number. **(B)** CM from osteoblasts cultured without chemokines enhanced TRACP^+^ MNC number. **(C)** CXCL8-CM enhanced osteoclastogenesis. IL-6 inhibition reduced this effect. **(D)** CCL20-CM enhanced osteoclastogenesis. IL-6 inhibition reduced this effect. **(E)** CM from osteoblasts cultured with CXCL8+CCL20 (20 pg/ml+50 pg/ml) enhanced osteoclastogenesis. IL-6 inhibition reduced this effect. **(F)** TNF-α-CM enhanced osteoclastogenesis (osteoclasts with >5 nuclei). IL-6 inhibition reduced this effect. Values are mean±SEM from 3 experiments, n = 9. Significant effect of chemokine, control-CM, and chemokine-CM, *p<0.05, **p<0.01, ***p<0.001. Significant effect of IL-6 inhibitor, ^**#**^p<0.05, ^**###**^p<0.001.

CXCL8 and CCL20 did not directly affect osteoclast formation (Fig 4A). In contrast, CM from osteoblasts cultured for 14 days increased osteoclast number by 1.5-fold (Fig 4B). CXCL8-CM increased the number of osteoclasts with 3–5 nuclei by 1.7-fold, and with >5 nuclei by 3.0-fold (Fig 4C). Inhibition of IL-6 nullified the stimulatory effect of IL-6 on the formation of osteoclasts with 3–5 nuclei, and reduced the formation of osteoclasts with >5 nuclei from 3-fold to 1.3-fold (Fig 4C). CCL20-CM increased the number of osteoclasts with 3–5 nuclei by 1.3-fold, and with >5 nuclei by 2.8-fold (Fig 4D). IL-6 inhibition nullified the stimulatory effect of IL-6 on the formation of osteoclasts with 3–5 nuclei, and reduced the formation of osteoclasts with >5 nuclei from 2.8-fold to 1.3-fold (Fig 4D). CXCL8+CCL20-CM increased the number of osteoclasts with 3–5 nuclei by 1.3-fold, and with >5 nuclei by 2.5-fold (Fig 4E). IL-6 inhibition nullified the stimulatory effect of IL-6 on the formation of osteoclasts with 3–5 nuclei, and reduced the formation of osteoclasts with >5 nuclei from 2.5-fold to 1.3-fold (Fig 4E). TNF-α-CM increased the number of osteoclasts with >5 nuclei by 3.7-fold, while IL-6 inhibition reduced the formation of osteoclasts with 3–5 nuclei from 3.7-fold to 1.4-fold (Fig 4F).

### CCL20-CM Enhanced Osteoclastic Bone Resorption

**Fig 5:**
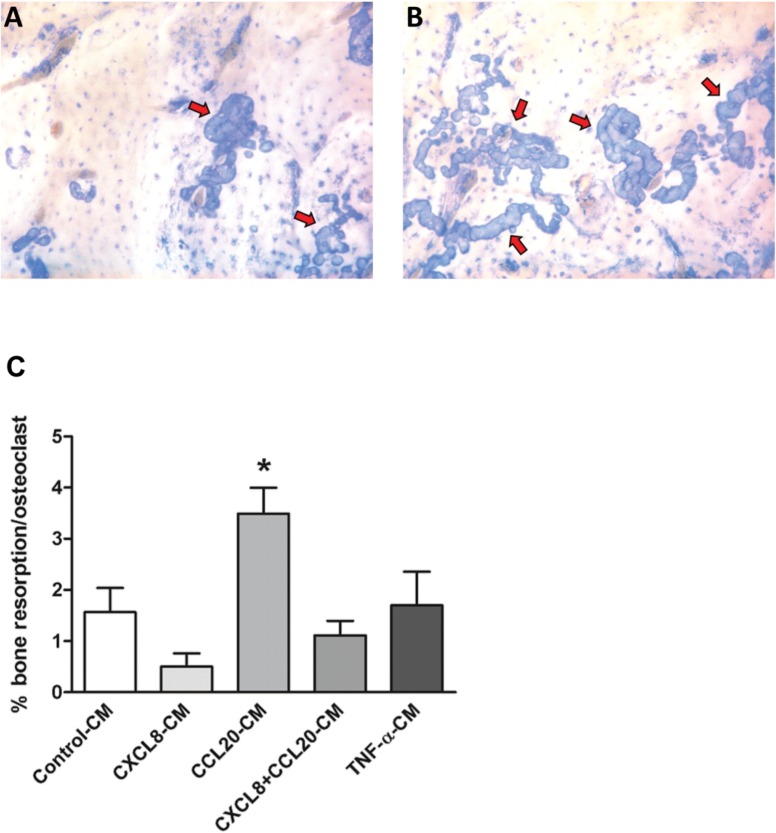
Effect of CM from osteoblasts cultured with CXCL8 (200 pg/ml) or CCL20 (500 pg/ml) on osteoclastic bone resorption. **(A)** Resorption pits (blue area; arrows) on a bone slice after 21 days of culture of PBMCs in the presence of control-CM. CXCL8-CM, CXCL8+CCL20-CM, and TNF-α-CM resulted in resorption pit formation, similar to those formed in the presence of control-CM (data not shown). **(B)** Resorption pits (blue area; arrows) on a bone slice after 21 days of culture of PBMCs in the presence of CCL20-CM. **(C)** CCL20 (500 pg/ml)-CM enhanced osteoclastic bone resorption compared to control-CM. CXCL8 (200 pg/ml)-CM, CXCL8 (20 pg/ml) +CCL20 (50 pg/ml)-CM, and TNF-α (100 ng/ml)-CM did not affect osteoclastic bone resorption. Values are mean±SEM from 3 experiments, n = 9. Significant effect of CM, *p<0.05.

Osteoclasts formed resorption pits on bovine cortical bone slices (Fig 5A and 5B). CCL20-CM enhanced osteoclastic bone resorption by 2.2-fold in comparison to control-CM (Fig 5C). CXCL8-CM, CXCL8+CCL20-CM, and TNF-α-CM did not affect osteoclastic bone resorption (Fig 5C).

## Discussion

In this study we analyzed whether chemokines potentially play a role in the emergence of osteoporosis in inflammatory diseases by studying their effect on osteoblast function and osteoblast-mediated osteoclast formation and activity. We found that CXCL8 and CCL20 did not inhibit osteoblast proliferation nor gene expression of the main matrix proteins *COL1*, *OPN*, and *OCN*. CXCL8 and CCL20 enhanced osteoblast-mediated osteoclastogenesis, partly via IL-6 production, suggesting that CXCL8 and CCL20 may contribute to generalized osteoporosis in RA by affecting bone cell communication.

The osteoblastic nature of human primary osteoblasts, as well as their osteocyte-like behavior has been shown earlier [28–31]. We found that these cells express functional chemokine receptors CXCR1 and CCR6, but not CXCR3, which is the receptor for CXCL9 and CXCL10. Lisignoli and colleagues reported that osteoblasts express functional CXCR3 [18]. This discrepancy in CXCR3 expression might be related to differences in anatomical location of the bone biopsies; we used biopsies from the iliac crest, and Lisignoli and colleagues from the tibial plateau [18].

Both CXCL8 and CCL20 enhanced gene expression of the cell proliferation marker *KI67* in osteoblasts. However we could not detect an effect of chemokines on osteoblast proliferation using the XTT assay or by measuring the total DNA content, which is in contrast with data published by others showing that CCL20 enhances proliferation of osteoblasts from RA patients [19]. This difference might be due to the fact that we obtained cells from patients without any signs of systemic inflammation, while Lisignoli and colleagues obtained cells from RA patients [19]. Our findings indicate that CXCL8 and CCL20 do not affect osteoblast proliferation.

Since proinflammatory cytokines inhibit osteoblast function [14, 32–34] we expected that CXCL8 or CCL20 would also inhibit osteoblast function. However we found that CXCL8 and CCL20 enhanced gene expression of some early osteogenic differentiation-related markers but did not affect P1NP production, ALP activity, nor gene expression of late osteogenic differentiation-related markers. CXCL8 and CCL20 increased *ALP* and *COL1* gene expression, but not P1NP and ALP activity, which might be due to altered post-transcriptional processing. In any case, our data suggests that CXCL8 and CCL20 do not negatively affect osteoblast function *in vitro*.

**Fig 6:**
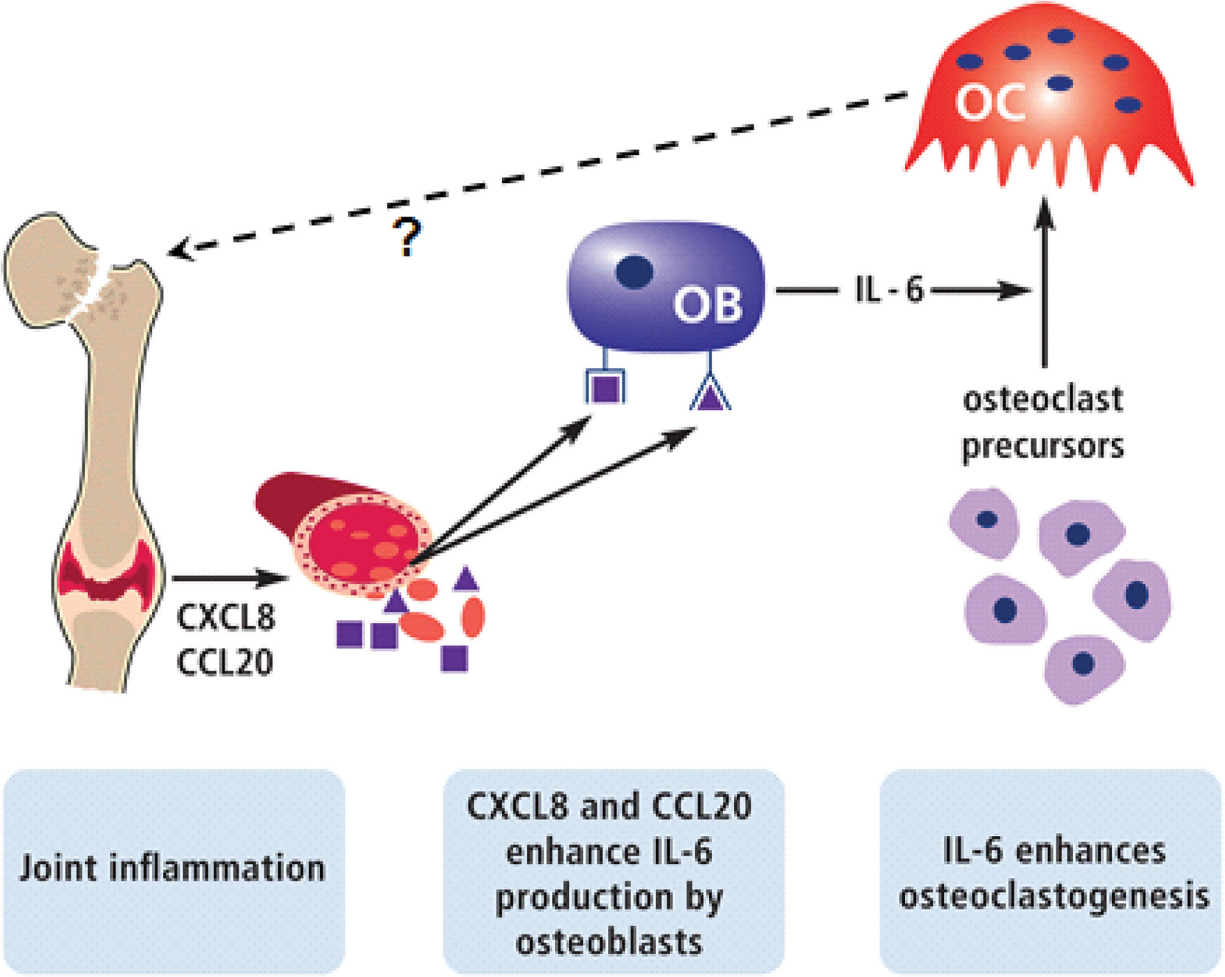
Pathophysiological model. Illustration of how CXCL8 and CCL20 might influence bone remodeling during systemic inflammation such as RA and might contribute to osteoporosis.

CXCL8 and CCL20 enhanced *IL-6* gene expression and protein production by osteoblasts. CXCL8 and CCL20 did not directly affect osteoclastogenesis, which is in accordance with findings by others [19, 20]. Here we show that CM from osteoblasts cultured for 14 days (control-CM; without chemokines) enhanced osteoclastogenesis *in vitro*, indicating that osteoblasts produce factors essential for osteoclast formation. Furthermore, our study reveals that CXCL8-CM, CCL20-CM, and TNF-α-CM enhanced osteoclastogenesis. CCL20-CM, but not CXCL8-CM, enhanced osteoclast activity. Osteoclast activity is not only regulated by the number of osteoclasts and tartrate-resistant acid phosphatase present in osteoclasts, but also by a number of other regulators such as cathepsin K, lysophosphatidic acid receptor type 1 (LPA1), lysosome associated membrane protein-2, and chloride channels CIC3 and CIC7 [35–38]. Osteoclast activity regulators might present differently in osteoclasts formed in the presence of CXCL8-CM compared with CCL20-CM, which might cause the difference in osteoclastic bone resorbing activity. We also found that CXCL8 and CCL20 enhanced *IL-6* gene expression and protein production by osteoblasts. Moreover IL-6 production was strongly enhanced by TNF-α treatment; a similar finding has been reported by Chaudhary et al. [9]. Inhibition of IL-6 robustly reduced the effect of CXCL8-CM, CCL20-CM, and TNF-α-CM on osteoclastogenesis. CCL20 has been suggested to play a role in the pathogenesis of rheumatoid arthritis [39]. IL-6 is one of the most potent stimulators of osteoclastic bone resorption and central to the pathogenesis of generalized osteoporosis in RA [40–42]. This corroborates with our data showing a stimulatory effect of CXCL8 and CCL20 on IL-6 production by osteoblasts, which enhanced osteoclastogenesis. CXCL8 and CCL20 might not only affect IL-6 production, but also the production of a whole cocktail of factors by osteoblasts. Therefore it is unlikely that IL-6 inhibition alone will completely block the effect of CXCL8 and/or CCL20 on osteoblast-mediated osteoclastogenesis. RANK, RANKL, and OPG are known key molecules involved in osteoclast formation and function [43]. In this study, RANKL gene expression by osteoblasts was below the detection limit, and therefore we did not test the effect of RANKL and OPG signaling molecules produced by osteoblasts on osteoclast formation. Our model uses exogenous recombinant RANKL to allow osteoclast formation, since osteoclast formation does not occur in the absence of RANKL, which created further limitation to analyze the role of RANKL and OPG in osteoclastogenesis. Detailed information on our model is provided in S1 Fig. Based on our findings, we created a pathophysiological model illustrating how CXCL8 and CCL20 might influence bone remodeling in inflammatory conditions and contribute to osteoporosis (Fig 6).

CXCL8 and CCL20 are abundantly present in synovial tissue, synovial fluid, and serum in RA, and their levels correlate with disease activity [8, 20, 44, 45]. Blockade of CXCL8 reduces inflammation in a murine RA model [46]. A CXCR1 antagonist has been shown to decrease clinical disease scores in a murine collagen-induced arthritis model [47]. A polymorphism of the CCR6 gene has been associated with RA susceptibility [48]. Moreover CCL20 has been suggested as an emerging player in the pathogenesis of rheumatoid arthritis [39]. Our study provides insight in the mechanism of action of CXCL8 and CCL20 with regard to the regulation of bone metabolism. Future studies in which CXCL8 and CCL20 are blocked could confirm whether CXCL8 and/or CCL20 might be new targets to prevent bone loss in inflammatory diseases such as RA. The combination of CXCL8 and CCL20 had a similar effect on osteoblast-mediated osteoclastogenesis as the individual CXCL8 and CCL20 at a 10-fold higher concentration. This indicates that the effect of CXCL8 and CCL20 on bone loss *in vivo* where combinations of cytokines and chemokines are present in the circulation, might be more pronounced than *in vitro*. A limitation of this study might be the relatively low number of patients included. Statistical significance between groups was not easily obtained due to the fairly large data variation, probably due to donor variation. Another limitation of this study is that the addition of exogenous recombinant RANKL (4 ng/ml) is essential for osteoclastogenesis in our experimental set up. This prevents analysis of the role of chemokine-induced osteoblast-mediated RANKL/OPG signaling on osteoclastogenesis. However, osteoblastic RANKL gene expression was below the detection level, even after treatment with CXCL8 or CCL20. Furthermore osteoblastic OPG expression was not affected by CXCL8 or CCL20. In this study IL-6 was a key molecule produced by osteoblasts as a result of CXCL8 and/or CCL20 treatment, which enhanced osteoclastogenesis and this effect of IL-6 was reduced by blocking IL-6.

In conclusion, our results indicate that CXCL8 and CCL20 did not significantly inhibit osteoblast proliferation and function, nor directly enhanced osteoclastogenesis. However, CXCL8 and CCL20 strongly enhanced osteoblast-mediated osteoclastogenesis, which seems partially mediated by CXCL8 and CCL20-induced IL-6 production by osteoblasts. Based on these findings, we speculate that CXCL8 and CCL20 may play a role in generalized osteoporosis during systemic inflammation in which serum levels of CXCL8 and CCL20 are elevated.

## Supporting Information

S1 Fig Schematic representation of experimental set up. (TIF)

S1 Table Primers used in the real-time PCR assay. (DOCX)
