# Supplementary figures and images for: CXCL8 and CCL20 Enhance Osteoclastogenesis via Modulation of Cytokine Production by Human Primary Osteoblasts
Source: PLoS One. 2015 Jun 23;10(6):e0131041. doi: 10.1371/journal.pone.0131041 (PMC4477884; doi:10.1371/journal.pone.0131041)

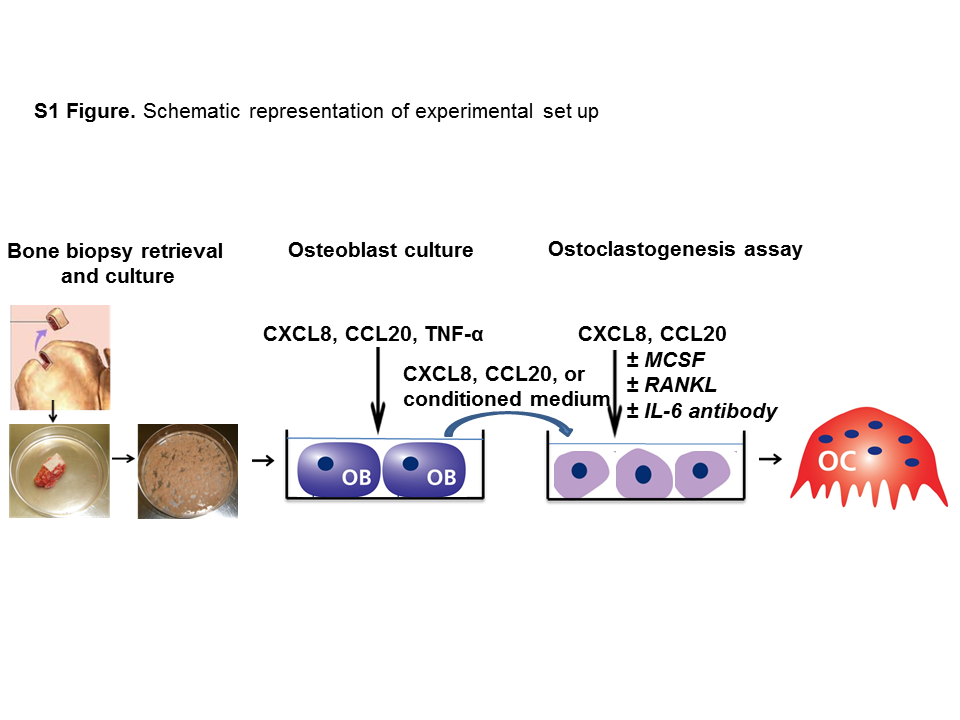

Supplement: S1 Fig — (TIF) [file pone.0131041.s001.tif]
