# Supplementary material for: CXCL8 and CCL20 Enhance Osteoclastogenesis via Modulation of Cytokine Production by Human Primary Osteoblasts
Source: PLoS One. 2015 Jun 23;10(6):e0131041. doi: 10.1371/journal.pone.0131041 (PMC4477884; doi:10.1371/journal.pone.0131041)
Supplement: S1 Table — (DOCX) [file pone.0131041.s002.docx]

**S1 Table.** Primers used in the real-time PCR assay.

| Gene | Oligonucleotide sequence | | Amplicon  length  (bp) |
| --- | --- | --- | --- |
| *YWHAZ* | Forward | 5' GATGAAGCCATTGCTGAACTTG 3' | 229 |
|  | Reverse | 5' CTATTTGTGGGACAGCATGGA 3' |  |
| *HPRT1* | Forward | 5' GCTGACCTGCTGGATTACAT 3' | 260 |
|  | Reverse | 5' CTTGCGACCTTGACCATCT 3' |  |
| *KI67* | Forward | 5' GGTGGGCACCTAAGACCTGAA 3' | 235 |
|  | Reverse | 5' TCCTAGGACTAGGAGCTGGAG 3' |  |
| *ALP* | Forward | 5' AGGGACATTGACGTGATCAT 3' | 242 |
|  | Reverse | 5' CCTGGCTCGAAGAGACC 3' |  |
| *COL1* | Forward | 5' TCCAACGAGATCGAGATCC 3' | 191 |
|  | Reverse | 5' AAGCCGAATTCCTGGTCT 3' |  |
| *OPN* | Forward | 5' TTCCAAGTAAGTCCAACGAAAG 3' | 181 |
|  | Reverse | 5' GTGACCAGTTCATCAGATTCAT 3' |  |
| *OCN* | Forward | 5' AGCCACCGAGACACCATGAGA 3' | 288 |
|  | Reverse | 5' CTCCTGAAAGCCGATGTGGTC 3' |  |
| *IL6* | Forward | 5' ACAGCCACTCACCTCTTCA 3' | 207 |
|  | Reverse | 5' ACCAGGCAAGTCTCCTCAT 3' |  |
| *IL1B* | Forward | 5' TGGAGCAACAAGTGGTGTTCT 3' | 270 |
|  | Reverse | 5' GAGAGGTGCTGATGTACCAGTT 3' |  |
| *RANKL* | Forward | 5' CATCCCATCTGGTTCCCATAA 3' | 60 |
|  | Reverse | 5'GCCCAACCCCGATCATG 3' |  |
| *M-CSF* | Forward | 5' CCGAGGAGGTGTCGGAGTAC 3' | 100 |
|  | Reverse | 5' AATTTGGCACGAGGTCTCCAT 3' |  |
| *OPG* | Forward | 5'TGGAATAGATGTTACCCTGTGTG 3' | 298 |
|  | Reverse | 5' GCTGCTCGAAGGTGAGGTTA 3' |  |
| *TNF-α* | Forward | 5' AGAGGGCCTGTACCTCATCT 3' | 315 |
|  | Reverse | 5' AGGGCAATGATCCCAAAGTAG 3' |  |
| *CYR61* | Forward | 5' CAACCCTTTACAAGGCCAGA 3' | 206 |
|  | Reverse | 5' TGGTCTTGCTGCATTTCTTG 3' |  |
| *CCL20* | Forward | 5' TGATGTCAGTGCTGCTACTC 3' | 142 |
|  | Reverse | 5' ATGTCACAGCCTTCATTGGC 3' |  |
| *IL17* | Forward | 5' CTACAACCGATCCACCTCAC 3' | 255 |
|  | Reverse | 5' ACAATCGGGGTGACACAGGT 3' |  |
| *CXCL8* | Forward | 5' TCTGCAGCTCTGTGTGAAG 3' | 147 |
|  | Reverse | 5' TGTGTTGGCGCAGTGTGG 3' |  |
